# Infective Endocarditis Associated with Atrial Septal Defect in an Intravenous Drug Abuser: A Case Report

**DOI:** 10.7759/cureus.2482

**Published:** 2018-04-15

**Authors:** Maha Jahangir, Marrium Nawaz, Fareha Jabbar, Fahad Khan, Nimra Hasnain

**Affiliations:** 1 Dow Medical College, Civil Hospital Karachi, Karachi, PAK; 2 Dow Medical College, Dow University of Health Sciences (DUHS), Karachi, PAK; 3 Department of Cardiology, Civil Hospital Karachi, Kara, PAK

**Keywords:** atrial septal defect (asd), intravenous drug abuse (ivda), infective endocarditis (ie), blood culture, methicillin-resistant staphylococcus aureus (mrsa)

## Abstract

Atrial septal defect (ASD) is a common congenital abnormality, which accounts for 20-40% of all the adult patients with congenital heart diseases. Due to the slow velocity of shunt flow, ASD has a negligible risk for infective endocarditis (IE). However, intravenous drug abuse (IVDA) is a potential cause for IE. IE remains a diagnostic and therapeutic challenge. Our case report demonstrates the atypical presentation of IE in an ASD patient. The diagnosis was made on the basis of modified Duke criteria, and blood cultures were found out to be positive for methicillin-resistant Staphylococcus aureus (MRSA). The treatment for IE was completed in six weeks with full recovery, and the patient underwent a surgery for ASD closure. This case highlights that IE should not be overlooked in ASD patients and that a high index of suspicion, in addition to proper antibiotic therapy, is lifesaving. Also, follow-up, along with rehabilitation measures, should be taken for patients with a history of drug abuse in order to prevent the risk of reinfection.

## Introduction

Atrial septal defect (ASD) accounts for approximately 20-40% of congenital heart diseases in adults. It is caused by an abnormality in the inter-atrial septum, producing left to right shunt predominantly [1]. Previously, studies have shown that ASD patients are not usually predisposed to infective endocarditis (IE) and the association of ASD with IE is quite rare [1-3]. Infective endocarditis is a serious condition that may be overlooked due to its less likely association with an atrial septal defect. We report the case of a 35-year-old male patient, diagnosed with ASD on a standard set of investigations, with IE suspected due to a persistent high-grade fever and intravenous drug abuse (IVDA).

## Case presentation

**Figure 1:**
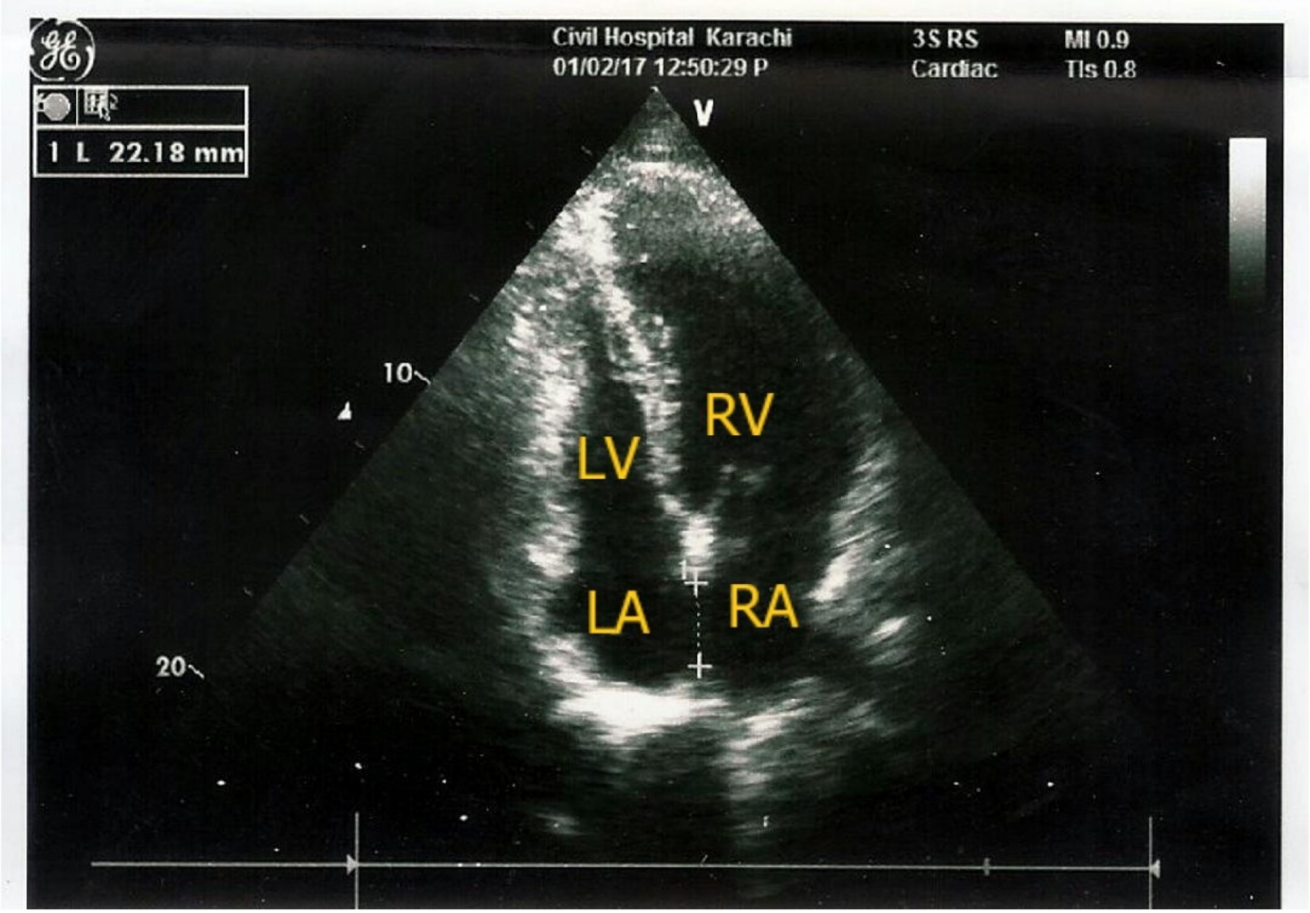
Apical four chamber view showing a large atrial septal defect (ASD)

A 35-year-old male presented to the Emergency Department of Civil Hospital Karachi with a 10-day history of high-grade fever, shortness of breath, palpitations, and joint pain. The patient had poor appetite and fatigue. He denied any history of trauma, allergies, any other medical conditions, or weight loss. A review of the cardiopulmonary, gastrointestinal, and genitourinary systems was unremarkable. He did not smoke or use alcohol, and there had been no changes in his daily routine. However, he was an intravenous drug abuser. The patient had no history of any major surgery. His history demonstrated an ostium secundum of 22.18 mm with left to right shunt, which was diagnosed previously by transoesophageal echocardiography (Figure 1). 

On physical examination, he was found to be dehydrated but oriented to time, place, and person. His blood pressure was 100/70 mm Hg and his heart rate was 100 beats/minute. The respiratory rate was 20 breaths/minute and his temperature was 102°F. His fever was associated with chills, rigors, and sweating with multiple joint pains. On cardiovascular examination, no murmur was auscultated. The lungs were clear on auscultation. The abdomen was non-tender; hepatomegaly or lymphadenopathy was not detected. However, his spleen was enlarged 2 cm below the costal margin. Motor and sensory examination of all four limbs were normal. Past and family history of the patient was insignificant.

The patient's workup was initiated. Laboratory findings included complete blood cell count (CBC) showing Hb level =  12.6g/dl (normal range: 13.0 - 16.5 gm/dL) and total leukocyte count (TLC) = 15,000/μL (normal range: 4 - 11 x 103/μL). Malarial parasite and dengue tests were insignificant. Other tests including serology for human immunodeficiency virus (HIV) and hepatitis showed normal findings. Due to his persistent high-grade fever, IE was suspected in the patient. Therefore, echocardiography and blood culture were performed.

Echocardiography did not show any vegetation. However, two blood samples drawn from two different sites (12 hours apart for culture) were found to be positive for methicillin-resistant Staphylococcus aureus (MRSA). The patient was treated with intravenous (IV) vancomycin, 1 gm once daily, and IV gentamicin, 80 mg twice a day, for a period of six weeks for S. aureus. The fever eventually subsided, and subsequently, a surgery for the closure of ASD was planned and executed successfully. The patient was discharged from the hospital after he recovered well from surgery. He was further referred to the rehabilitation centre for drug abuse treatment. The patient visits on a monthly basis for follow–up and continues to do well.

## Discussion

Patients with congenital heart defects have 15 - 140 times higher risk of developing IE than the general population [2].  However, as compared to the other congenital heart defects, the association of  ASD with IE is rare [2] since the anatomic localization of IE in ASD is only 0.4% [3]. Over an observational period of 18 years, Li et al. reported no incidence of IE in 5,000 adult congenital heart disease patients before or after ASD surgery [4].

IE remains a diagnostic and therapeutic challenge. If left untreated, it is generally fatal. Our patient presented with a major criterion and three minor criteria, thus satisfying the modified Duke criteria [5] for definite endocarditis. Minor criteria met included fever > 38ºC, splenomegaly, predisposing congenital heart disease, and drug abuse, while the major criterion was two positive blood cultures of S. aureus drawn 12 hours apart. Blood culture remains a cornerstone for diagnosis of IE. S. aureus is currently the leading pathogen causing acute IE [3, 5]  with an overall mortality rate of 22 - 34% [6].

Intravenous drug abuse is one of the leading causes of IE due to S. aureus. We speculate that IVDA was the most likely cause of developing IE in our patient. The patient presented with arthralgia, which is the most frequent but often overlooked complaint in IE due to IVDA, with S. aureus being the most common offender [7].

For intravenous drug users, two weeks of therapy with nafcillin, plus an aminoglycoside, is generally effective [8]. However, since our patient had an MRSA strain, IV vancomycin, together with IV gentamicin, was given for a span of six weeks to which he promptly responded and his fever subsided within six weeks of treatment. As IE poses a high mortality risk, it is emphasized that vancomycin therapy should be initiated as soon as the blood cultures are available. Due to the risk of cardiac overload, early surgical closure of ASD is necessary in order to avoid fatal complications. Therefore, surgery was planned and executed with the patient’s consent. Considering the possibility of reinfection in IVDA, patients should be referred to the rehabilitation centre for further drug abuse treatment and prevention.

## Conclusions

IVDA is a common cause of IE. Hence, IE should be considered in patients presenting with IVDA with various clinical scenarios. However, ostium secundum is a rare site for clinicians to suspect and hence, explore in case of infective endocarditis. A high degree of suspicion is required with unexplained fever and presentation in patients with IVDA. Better clinical and diagnostic techniques, such as serology, additional blood cultures, or newer histological and molecular techniques and advancement in technologies, combined with close cooperation of a cardiologist, cardiac surgeon, and an infectious disease physician, can help ensure optimal diagnosis, management, and solve this diagnostic dilemma.
